# Evaluating the Effects of *Crocus sativus* L. Herbal Product on Chronic Fatigue Syndrome in Patients with Chronic Obstructive Pulmonary Disease: A Randomized, Double-Blind, Placebo-Controlled Clinical Trial

**DOI:** 10.5812/ijpr-165333

**Published:** 2025-10-29

**Authors:** Farzaneh Dastan, Jamshid Salamzadeh, Jalal Heshmatnia, Reza Mahmoudian

**Affiliations:** 1 Department of Pharmacy, Monash Health Center, Melbourne, Australia; 2 Department of Clinical Pharmacy, School of Pharmacy, Shahid Beheshti University of Medical Sciences, Tehran, Iran; 3 Chronic Respiratory Diseases Research Center, National Research Institute of Tuberculosis and Lung Diseases (NRITLD), Shahid Beheshti University of Medical Sciences, Tehran, Iran

**Keywords:** Chronic Obstructive Pulmonary Disease (COPD), Saffron, Fatigue, Chronic Fatigue Syndrome, *Crocus sativus*

## Abstract

**Background:**

Chronic fatigue syndrome (CFS) is a complex and debilitating disorder characterized by extreme fatigue that cannot be explained by any underlying medical condition. Chronic fatigue syndrome significantly impacts the daily lives of patients with chronic obstructive pulmonary disease (COPD). Currently, there is no definitive treatment for CFS.

**Objectives:**

This study aimed to determine the effects of *Crocus sativus* L. herbal product on improving CFS and quality of life (QOL) in patients with COPD.

**Methods:**

This study was conducted as a randomized, double-blind, placebo-controlled clinical trial. Chronic obstructive pulmonary disease patients with CFS were randomly assigned to either the intervention or control group using block randomization generated by the Sealed Envelope online tool. Data were coded to ensure that both researchers and analysts were blinded to group assignments. Participants in the intervention group (n = 37) received 30 mg of dry saffron extract capsules twice daily for eight weeks, while those in the control group (n = 34) received a placebo. The primary outcome was the effect of *C. sativus* on CFS, assessed using the Chronic Respiratory Questionnaire (CRQ) and the Manchester COPD Fatigue Scale (MCFS). The secondary outcome was the effect on QOL, measured using the St. George’s Respiratory Questionnaire (SGRQ). Patients were evaluated at baseline, after one month, and after two months.

**Results:**

Based on mixed repeated measures ANOVA models, the effect of *C. sativus* on CRQ total scores was significant (P < 0.001), while the effect on the Dyspnea subscale was not significant (P = 0.38). The effect on chronic fatigue status based on MCFS was significant (P < 0.001). The effect on total QOL score based on SGRQ was also significant (P = 0.012), while the effect on the Symptoms subscale was not significant (P = 0.158).

**Conclusions:**

The findings indicate that *C. sativus* herbal product is effective in reducing CFS and improving QOL in COPD patients. However, it may not be effective in reducing COPD symptoms, including dyspnea.

## 1. Background

Chronic fatigue syndrome (CFS) is a complex and debilitating disorder characterized by extreme fatigue that cannot be explained by any underlying medical condition (1, 2). Chronic fatigue syndrome affects daily functioning (3), social interactions (4), quality of life (QOL) (5), and other aspects of life. Fatigue is a common, often temporary feeling of tiredness or lack of energy that usually improves with rest. In contrast, CFS is a complex, long-lasting condition characterized by severe fatigue lasting more than six months, which is not relieved by rest and significantly impairs daily functioning. Chronic fatigue syndrome also involves additional symptoms like post-exertional malaise, unrefreshing sleep, and cognitive difficulties (6). The global prevalence of CFS varies widely, ranging from 0.01% to 7.62% (7). However, most studies estimate it to be between 0.2% and 1% (8, 9). In Iran, accurate statistics on CFS prevalence are lacking, but two studies reported rates of 11.4% among medical students in Guilan city (10) and 14.1% among a sample of nurses (11). Chronic obstructive pulmonary disease (COPD) is a common, progressive, preventable, and treatable disease characterized by persistent respiratory symptoms and airflow limitation (12). A comprehensive review of 26 studies estimated the global COPD prevalence at 7.6% (95% CI: 5.5% - 20%) (13), with earlier estimates ranging from 2% to 12% reported by the global burden of disease (GBD) and other sources (14). Chronic obstructive pulmonary disease patients often experience impaired QOL due to breathlessness, chronic cough, sputum production, reduced exercise tolerance, and frequent exacerbations that negatively impact both physical and emotional well-being (15, 16). It results in 3.3 million deaths and 74.4 million disability-adjusted life years (DALYs) (17), ranking as the third leading cause of death (18). Chronic obstructive pulmonary disease is also a major public health problem worldwide (18), imposing a significant economic burden of $2.1 trillion in 2010, projected to double by 2030, primarily driven by hospitalizations from exacerbations (19), and straining medical resources (12). *Crocus sativus* L. (Iridaceae), commonly known as saffron, is a perennial stemless herb that is widely cultivated in Iran and other countries such as India and Greece. Commercial saffron comprises the dried red stigma with a small portion of the yellowish style (20). In view of its wide range of medical uses, saffron has undergone extensive phytochemical and biochemical studies, and a variety of biologically active ingredients have been isolated. Characteristic components of saffron are crocin (responsible for the color), Picrocrocin (responsible for the bitter taste), and Safranal (responsible for odor and aroma) (20). In previous investigations, crocin supplementation has been shown to effectively restore the oxidant/antioxidant balance and improve inflammatory conditions in patients with COPD. This improvement is associated with enhanced performance in the 6-minute walking distance test (6MWD) and increased exercise capacity in these patients (21). Chronic fatigue syndrome significantly impacts the daily lives of patients with COPD, exacerbating physical, psychological, and social burdens (22). The multidimensional nature of fatigue, encompassing both physical and mental aspects, complicates COPD management by affecting patients' ability to engage in physical activity and social interactions, leading to social restrictions and poor QOL (23). Fatigue is a highly prevalent symptom among COPD patients, with prevalence rates ranging from 48.5% to 88.62% (24-26), and it has a significant negative impact on health-related QOL (27). Patients describe fatigue as severely impairing their daily functioning (28), emphasizing the need for targeted interventions to effectively manage this debilitating symptom.

## 2. Objectives

Standard treatments for CFS have traditionally included cognitive behavioral therapy (CBT) and graded exercise therapy (GET), which have been recommended by health authorities such as the US Centers for Disease Control and the UK NICE guidelines (29). However, the efficacy and safety of these treatments remain subjects of debate. While some studies and reviews suggest that CBT and GET can improve fatigue and physical function (30-34), other research highlights significant patient distress and potential harm, with many patients reporting worsened symptoms following GET (35, 36). Additionally, the effectiveness of these therapies appears modest, and adherence rates are often low, indicating poor tolerance among patients (37, 38). Regarding these treatments, limitations exist. Current therapies for CFS and COPD are limited and often ineffective. Chronic obstructive pulmonary disease treatments such as bronchodilators and corticosteroids manage symptoms but do not halt disease progression (2, 39), while CFS treatments fail to address the complex pathophysiology, resulting in inadequate symptom relief and functional improvement (40). *Crocus sativus*, particularly its compound crocin, has demonstrated therapeutic potential in improving pulmonary function tests, exercise capacity, and reducing inflammatory markers in COPD (21, 41). Additionally, this plant has been studied for its anti-inflammatory, antioxidant, and anti-carcinogenic properties, making it a potential candidate for treating various diseases (42). Its extracts have also been explored for medicinal use via inhalation delivery mechanisms, indicating its potential for treating different conditions (43). Although more extensive trials are needed in CFS, existing research suggests that *C. sativus* may be effective in managing CFS and other diseases. The purpose of this research is to investigate the effect of the *C. sativus* herbal product on CFS and QOL in patients with COPD.

## 3. Methods

### 3.1. Study Design and Setting

This study was designed as an open-label, randomized, double-blind clinical trial conducted at the lung clinic of Masih Daneshvari Hospital, affiliated with Shahid Beheshti University of Medical Sciences, Tehran, Iran. The study was registered with the Ethics Committee of the Shahid Beheshti School of Pharmacy (IR.SBMU.PHARMACY.REC.1402.033) and the Iranian Registry of Clinical Trials (IRCT20151227025726N34).

### 3.2. Patients

Patients referred to the lung clinic of Masih Daneshvari Hospital were considered eligible if they met the following inclusion criteria: Age over 18 years, a confirmed diagnosis of both COPD according to global initiative for chronic obstructive lung disease (GOLD) guidelines (44) and CFS based on Institute of Medicine (IOM) 2015 Criteria (6). Exclusion criteria included: Pregnancy or breastfeeding; rheumatoid diseases or osteoarthritis; electrolyte imbalances; adrenal insufficiency; obstructive sleep apnea; acute exacerbation of COPD within the past month; history of central nervous system disease; current use of sleep-sedative drugs, stimulants, antidepressants, or anticoagulants; known allergy to saffron or any components of the formulation; and any diagnosed neurological or psychiatric illness. To date, no previous trials have investigated the effects of saffron or other herbal extracts on CFS symptoms in COPD patients, making it difficult to reliably estimate the effect size for the primary outcome measure. Therefore, this trial utilized a convenience sample of 76 adults.

### 3.3. Randomization and Patient Allocation

Block randomization was used in this study. Thirty-eight blocks of two patients each were generated using an online tool (https://www.sealedenvelope.com/simple-randomiser/v1/lists). In each block, one patient was assigned to the *C. sativus* group and the other to the control group. Both the researchers and data analysts were blinded to group allocation and only received coded data.

### 3.4. Study Protocol

Baseline data included sociodemographic information, medical and medication history, and assessment of dyspnea using the Modified Medical Research Council (mMRC) scale. Patients in the intervention group received capsules containing 30 mg of dry saffron extract (produced by Faran Shimi Company) that was standardized based on 2% safranal, administered twice daily for two months. This dosage schedule and treatment duration were selected based on previous studies (21, 45, 46). The control group received a matching placebo following the same regimen. The placebo capsule contains all the components of the drug formulation except the active ingredient, saffron extract. The excipients, including microcrystalline cellulose, magnesium stearate, hard gelatin capsule, and silicon dioxide, were used in both the drug and placebo groups. The primary outcome was the effect of *C. sativus* on CFS symptoms in COPD patients, assessed using the Chronic Respiratory Questionnaire (CRQ) and the Manchester COPD Fatigue Scale (MCFS). The secondary outcome was the impact on health-related QOL, measured by the St. George’s Respiratory Questionnaire (SGRQ). All outcomes were evaluated at baseline and at multiple follow-up points over 8 weeks. Chronic Respiratory Questionnaire and MCFS were translated into Persian using a standardized forward-backward translation procedure. Two independent bilingual translators performed the forward translation from English to Persian, followed by backward translation into English by two other bilingual experts. A panel of specialists, including pulmonologists and linguists, reviewed all versions to resolve discrepancies and ensure conceptual equivalence. The final Persian versions of CRQ and MCFS were pilot-tested in a sample of 20 COPD patients to assess internal consistency. Cronbach’s alpha coefficients for both questionnaires exceeded 0.7, indicating acceptable reliability. Content validity was established through expert review. The Persian version of the SGRQ has been previously validated (47).

### 3.5. Chronic Respiratory Questionnaire

Chronic Respiratory Questionnaire consists of 20 items covering four distinct domains: Dyspnea, fatigue, emotional function, and mastery. The dyspnea domain is unique, as it asks participants to identify the five daily activities that most trigger their dyspnea and then rate the severity associated with each activity. This personalized approach tailors the assessment to individual experiences. All CRQ items are rated on a 7-point scale measuring function or perception. Domain scores are calculated by averaging the individual item scores within each domain (48).

### 3.6. Manchester Chronic Obstructive Pulmonary Disease Fatigue Scale

Manchester COPD Fatigue Scale is a self-reported instrument that assesses fatigue experienced by patients with COPD over the past two weeks. It consists of 27 items, each scored on a 5-point scale with values of 0, 0.5, 1, 1.5, and 2. The total MCFS score ranges from 0 to 54, with higher scores indicating greater levels of fatigue. This scale has demonstrated reliability and validity in measuring overall fatigue in patients with COPD (49).

### 3.7. St. George’s Respiratory Questionnaire

The SGRQ measures health-related quality of life (HRQoL) in patients with asthma or COPD (50). It consists of 50 items and yields four scores — symptoms, activity, impacts, and total — ranging from 0 to 100, with 100 indicating the worst condition.

### 3.8. Statistical Analysis

Between-group differences in outcomes were assessed using Mixed Repeated Measures ANOVA with Greenhouse–Geisser adjustment. A two-sided P-value < 0.05 was considered statistically significant.

## 4. Results

**Figure 1.**
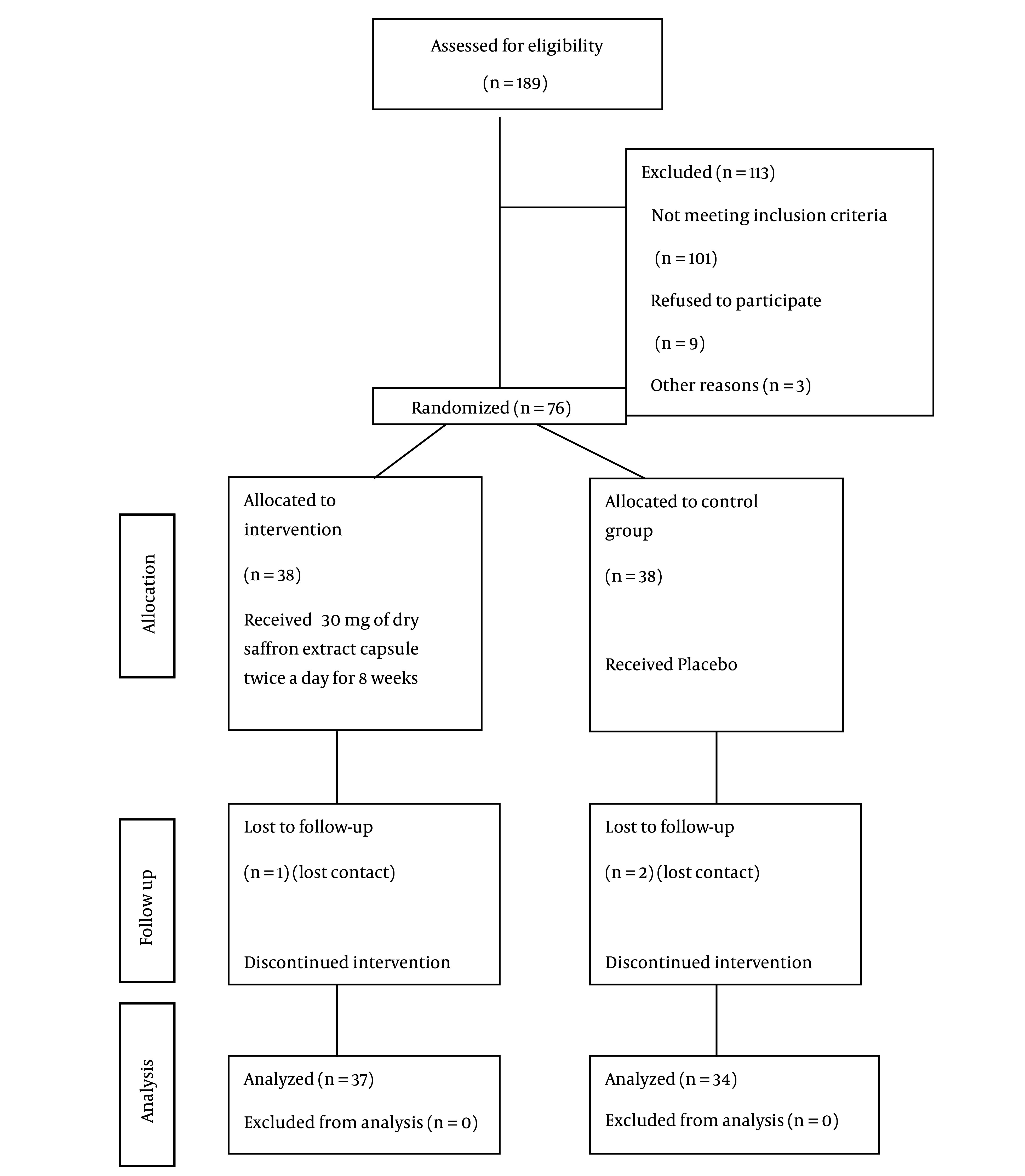
CONSORT diagram: Enrollment, randomization, and allocation of patients participating in the study

This study was conducted from October 2023 to September 2024. Of the initial 189 patients, 76 were randomized. Five patients were excluded due to lost contact or COPD exacerbation — four from the control group and one from the intervention group. The final statistical analysis included a total of 71 patients (Figure 1). No side effects were reported in either the intervention or placebo groups.

Table 1 presents the demographic characteristics of the patients in both the intervention and control groups. Additionally, the comparison of mMRC scores between the groups showed no statistically significant difference (P = 0.327). Comorbidities, such as hypothyroidism, heart failure, hypertension, diabetes, liver disease, and other medical conditions, were documented for all patients. Analysis showed that the prevalence of these comorbidities did not differ significantly between the intervention and control groups.

**Table 1.** Baseline Characteristics of Patients in Intervention and Control Groups ^a^

Variables	Intervention (n = 37)	Control (n = 34)	P-Value
**Age (y)**	63.8 ± 8.2	64.0 ± 9.6	0.89
**Gender**			0.494
Male	35 (94.6)	34 (100)	
Female	2 (5.4)	0 (0)	
**Height (cm)**	171.3 ± 7.0	171.3 ± 7.1	0.86
**Weight (kg)**	63.2 ± 14.8	69.6 ± 16.8	0.093
**Comorbidities**			0.322
With comorbidity	18 (48.6)	9 (26.5)	
Without comorbidity	19 (51.4)	25 (73.5)	

^a^ Values are presented as mean ± SD or No. (%).

Assessment of the effect of *C. sativus* herbal product on chronic fatigue (total scores and subscales) based on the CRDQ/CRQ criteria. Based on the Mixed Repeated Measures ANOVA models, the effect of the *C. sativus* herbal product on the total CRQ fatigue scores (P < 0.001), and the subscales of mastery (P < 0.001), fatigue (P < 0.001), and emotional (P < 0.001) was significant, while on the subscale of dyspnea was non-significant (P = 0.38) (Table 2). 

**Table 2.** Status of Chronic Fatigue Based on CRQ (Total Scores and Each of its Sub-scales) by Group ^a^

Group and Time (mo)	Total Score	Fatigue Score	Emotional Score	Mastery Score	Dyspnea Score
**Intervention**					
0	3.42 ± 0.18 (3.06 - 3.78)	2.92 ± 0.18 (2.56 - 3.28)	3.70 ± 0.20 (3.30 - 4.10)	3.87 ± 0.21 (3.46 - 4.28)	3.15 ± 0.31 (2.54 - 3.76)
1	4.04 ± 0.18 (3.68 - 4.40)	3.90 ± 0.19 (3.52 - 4.28)	4.64 ± 0.19 (4.26 - 5.02)	4.42 ± 0.19 (4.04 - 4.80)	3.31 ± 0.31 (2.70 - 3.92)
2	4.23 ± 0.19 (3.85 - 4.61)	4.04 ± 0.20 (3.64 - 4.44)	4.82 ± 0.20 (4.42 - 5.22)	4.64 ± 0.20 (4.24 - 5.04)	3.39 ± 0.30 (2.80 - 3.98)
**Placebo**					
0	3.97 ± 0.19 (3.59 - 4.35)	3.13 ± 0.19 (2.75 - 3.51)	4.31 ± 0.21 (3.89 - 4.73)	4.36 ± 0.22 (3.92 - 4.80)	3.68 ± 0.32 (3.06 - 4.30)
1	4.13 ± 0.19 (3.75 - 4.51)	3.30 ± 0.20 (2.90 - 3.70)	4.50 ± 0.20 (4.10 - 4.90)	4.40 ± 0.20 (4.00 - 4.80)	3.85 ± 0.32 (3.23 - 4.47)
2	4.21 ± 0.20 (3.81 - 4.61)	3.39 ± 0.21 (2.97 - 3.81)	4.45 ± 0.21 (4.03 - 4.87)	4.53 ± 0.21 (4.11 - 4.95)	3.82 ± 0.32 (3.20 - 4.44)

^a^ Values are presented as mean ± SE (95% CI).

Assessment of the effect of *C. sativus* herbal product on chronic fatigue using the MCFS criteria. According to the results of the mixed repeated-measures ANOVA, the effect of the herbal product on chronic fatigue status, as measured by the MCFS criteria, was statistically significant (P < 0.001). The adjusted mean scores for chronic fatigue status are presented in Table 3, categorized by group and time point.

**Table 3.** Status of Chronic Fatigue Using the Manchester Chronic Obstructive Pulmonary Disease Fatigue Scale Criteria

Group and Time (mo)	Mean ± SE (95% CI)
**Intervention**	
0	37.19 ± 1.81 (33.64 - 40.73)
1	31.24 ± 1.73 (27.86 - 34.63)
2	29.93 ± 1.82 (26.37 - 33.50)
**Placebo**	
0	32.79 ± 1.89 (29.10 - 36.49)
1	30.53 ± 1.80 (26.99 - 34.06)
2	31.40 ± 1.90 (27.68 - 35.12)

Assessment of the effect of *C. sativus* herbal product on QOL using SGRQ criteria. Based on Mixed Repeated Measures ANOVA models and the SGRQ criteria, the *C. sativus* herbal product had a statistically significant effect on the total QOL score (P = 0.012), as well as on the Activity (P = 0.027) and Impact (P = 0.012) subscales. However, its effect on the Symptoms subscale was not significant (P = 0.158). The adjusted mean scores for the total QOL and its subscales are presented in Table 4, categorized by group and time of measurement.

**Table 4.** Quality of Life Status (Total Scores and Subscales) by Group According to St. George’s Respiratory Questionnaire Criteria ^a^

Group and Time (mo)	Total Score	Activity Score	Impact Score	Symptom’s Score
**Intervention**				
0	56.74 ± 2.73 (51.37 - 62.11)	74.70 ± 3.85 (67.14 - 82.26)	45.35 ± 2.87 (39.73 - 50.97)	60.34 ± 2.75 (54.96 - 65.72)
1	50.50 ± 4.47 (42.98 - 58.02)	68.89 ± 3.63 (61.84 - 75.94)	39.68 ± 2.50 (35.03 - 44.33)	53.51 ± 2.69 (48.16 - 58.86)
2	48.22 ± 2.46 (43.38 - 53.06)	68.66 ± 4.49 (60.07 - 77.25)	35.38 ± 2.50 (30.73 - 40.03)	53.25 ± 2.67 (47.93 - 58.57)
**Placebo**				
0	47.72 ± 2.89 (42.05 - 53.39)	62.53 ± 4.01 (54.64 - 70.42)	37.63 ± 3.00 (31.74 - 43.52)	57.83 ± 2.87 (52.16 - 63.50)
1	45.46 ± 2.62 (40.32 - 50.60)	60.87 ± 3.79 (53.45 - 68.29)	35.68 ± 2.61 (30.47 - 40.89)	54.81 ± 2.81 (49.29 - 60.33)
2	44.55 ± 2.60 (39.44 - 49.66)	62.01 ± 3.64 (55.01 - 69.01)	34.53 ± 2.61 (29.32 - 39.74)	52.50 ± 2.79 (47.05 - 57.95)

^a^ Values are presented as mean ± SE (95% CI).

## 5. Discussion

This study investigated the effect of a *C. sativus* herbal product on chronic fatigue and QOL in patients with COPD. The results demonstrate significant improvements in both chronic fatigue and QOL among COPD patients treated with the herbal intervention. Specifically, the intervention group showed a marked reduction in total chronic fatigue scores at multiple time points, as measured by the CRQ and the MCFS. The total CRQ scores in the intervention group were significantly lower than those in the placebo group, with an effect size of Eta-squared = 0.166 (P < 0.001). This reduction was consistently observed across several subscales, including mastery (P < 0.001), fatigue (P < 0.001), and emotional function (P < 0.001). However, the dyspnea subscale did not show a significant difference between groups (P = 0.38), suggesting that while the *C. sativus* herbal product effectively reduces general fatigue and enhances emotional and psychological well-being, it may not have a significant impact on dyspnea-specific symptoms. Further analysis using the MCFS reinforced these findings. The intervention group demonstrated a significant reduction in chronic fatigue status (P < 0.001), with adjusted mean scores indicating a consistent decline in fatigue levels throughout the study period.

Regarding QOL, significant improvements were observed in the intervention group’s scores on the SGRQ. The total QOL score improved significantly (P = 0.012), with notable gains in the activity (P = 0.027) and impact (P = 0.012) subscales. These results suggest that the herbal product enhances patients’ ability to perform daily activities and reduces the overall impact of COPD on their lives. However, the symptoms subscale did not show a significant improvement (P = 0.158), indicating that the product’s benefits may be more pronounced in functional and emotional domains rather than directly alleviating respiratory symptoms.

Our study had more male participants, reflecting the higher prevalence of COPD among men in Iran, largely due to greater smoking and occupational exposures (51). The study’s results align with existing research that highlights the efficacy of alternative treatments in managing chronic fatigue and improving QOL in both COPD and CFS. For instance, a review of clinical trials in CFS underscored the need for effective treatments beyond traditional approaches such as CBT and GET, which have shown mixed results and low adherence rates due to patient distress (29-33, 35-38). The findings of this study add to the growing body of evidence supporting the use of alternative therapies, including herbal products, in managing chronic conditions.

In contrast, standard treatments for COPD such as bronchodilators and corticosteroids primarily target lung function improvement and symptom management (52-54). Non-pharmacological interventions like pulmonary rehabilitation and patient education also play vital roles in enhancing outcomes and QOL in COPD patients. However, these conventional treatments often do not adequately address fatigue and the emotional burden experienced by patients, underscoring the importance of exploring alternative therapies like *C. sativus*.

The results of this study, demonstrating significant reductions in chronic fatigue and improvements in QOL among COPD patients treated with the *C. sativus* herbal product, are consistent with existing literature on the anti-inflammatory and antioxidant effects of crocin in COPD. Previous clinical trials, such as that by Ghobadi et al., found that crocin supplementation effectively decreased inflammatory markers, including NF-κB and total oxidant status (TOS), while increasing total antioxidant capacity (TAOC). This biochemical modulation was accompanied by improvements in exercise capacity, measured by the 6MWD, and enhanced pulmonary function tests — both critical factors influencing QOL in COPD patients (21). Similarly, these findings support the hypothesis that crocin’s antioxidant and anti-inflammatory mechanisms contribute to the observed reduction in fatigue scores reported in this study. However, our study did not demonstrate any improvement in shortness of breath symptoms in the intervention group compared to the placebo group.

Ashtiani et al. conducted a pre-post study investigating the effects of a simple saffron syrup on fatigue in patients with multiple sclerosis (MS). In their study involving 30 participants, patients consumed 7.5 cc of saffron syrup every eight hours for two months. Fatigue severity, assessed using the Fatigue Severity Scale (FSS), showed a significant reduction following the intervention (P < 0.001), indicating that saffron syrup effectively alleviated fatigue symptoms in MS patients. Importantly, the treatment was well-tolerated, with no significant side effects reported. These results suggest that saffron syrup may serve as a safe and effective adjunct therapy to reduce fatigue — a major and debilitating symptom of MS — thereby improving patients’ QOL (45). The anti-fatigue effects are believed to be mediated by crocin’s potent antioxidative and anti-inflammatory properties, which reduce oxidative stress and systemic inflammation — key contributors to fatigue development, especially in chronic neurological and inflammatory conditions (55). Neuroprotective effects observed in animal models further support saffron’s role in alleviating cognitive and physical fatigue by enhancing neurological function and reducing neuroinflammation (56).

Mirzaei et al. conducted a randomized clinical trial to evaluate the safety and efficacy of Jollab, a tonic beverage containing saffron, honey, and rose water, for managing cancer-related fatigue in breast cancer patients. Seventy-five patients were randomly assigned to receive either Jollab or a placebo three times daily for four weeks. Fatigue was assessed at baseline and post-intervention using the Visual Analogue Fatigue Scale (VAFS), FSS, and Cancer Fatigue Scale (CFS). The results demonstrated a significant reduction in fatigue scores in the Jollab group on both the VAFS and FSS (P = 0.000), with improvements also observed in the physical and cognitive subscales of the CFS. However, no significant change was noted in the affective subscale in either group (46). Moreover, crocin has been reported to modulate neurotransmitter systems, including dopaminergic, noradrenergic, and serotonergic pathways, which likely contribute to improved mood and the alleviation of fatigue-related mental symptoms (57).

In summary, saffron and crocin appear to reduce fatigue by enhancing antioxidant defenses, suppressing inflammatory signaling pathways, protecting neural function, and modulating mood-related neurotransmitters, making saffron a promising complementary agent for fatigue management in chronic diseases. We included participants over 18 years old without an upper age limit to improve the generalizability of our findings to adults with COPD and CFS. Since COPD is common in older adults, excluding them could limit the applicability of the results. We also monitored all participants closely for any age-related adverse events to ensure safety. The study’s double-blind, randomized, placebo-controlled design strengthens the reliability of the findings. The use of validated measurement tools including the CRQ, MCFS, and SGRQ ensures a comprehensive and robust assessment of chronic fatigue and QOL.

However, several limitations should be acknowledged. The relatively short duration, with outcomes assessed at three time points over two months, restricts insight into the long-term sustainability of the observed benefits. Moreover, the specific mechanisms by which *C. sativus* influences chronic fatigue and QOL remain unclear, warranting further biochemical and physiological investigation. Additionally, the relatively small sample size and focus solely on COPD patients limit the generalizability of the results. Future research should involve larger and more diverse populations to validate these findings across different settings. Finally, the reliance on self-reported measures introduces the possibility of reporting bias, highlighting the need for incorporating objective assessments or biomarkers in subsequent studies.

### 5.1. Conclusions

The findings of this study suggest that the *C. sativus* herbal product may be effective in reducing chronic fatigue and improving QOL in patients with COPD. Significant improvements in fatigue scores and several quality-of-life subscales indicate the potential of this herbal intervention to address aspects of COPD beyond respiratory symptoms alone. However, given the limitations of our study, further research is needed to confirm these results, clarify the underlying mechanisms, and assess the long-term benefits of *C. sativus* in larger and more diverse patient populations. Such studies will be important to better understand the therapeutic potential and clinical applicability of this herbal product in COPD and possibly other chronic diseases.

## Data Availability

The data presented in this study are uploaded during submission as a supplementary file and are openly available for readers upon request.

## Clinical Trial Registration Code:

IRCT20151227025726N34.
